# Polymorphisms in *CYP1B1*, *CYP3A5*, *GSTT1*, and *SULT1A1* Are Associated with Early Age Acute Leukemia

**DOI:** 10.1371/journal.pone.0127308

**Published:** 2015-05-18

**Authors:** Bruno Almeida Lopes, Mariana Emerenciano, Bruno Alves Aguiar Gonçalves, Tállita Meciany Vieira, Ana Rossini, Maria S. Pombo-de-Oliveira

**Affiliations:** 1 Pediatric Hematology-Oncology Program, Research Center, Instituto Nacional de Câncer, Rio de Janeiro, RJ, Brazil; 2 Department of Biochemistry, Universidade do Estado do Rio de Janeiro, Rio de Janeiro, RJ, Brazil; National Center for Toxicological Research, US Food and Drug Administration, UNITED STATES

## Abstract

Based on observational studies, early age leukemia (EAL) was associated with maternal hormone exposure during pregnancy. We studied the association between genetic polymorphisms of estrogen metabolism and EAL. Using data from the Brazilian Collaborative Study Group of Infant Acute Leukemia (2000–2012), 350 cases and 404 age-matched controls and 134 mothers of cases and controls were genotyped to explore polymorphisms in genes of the estrogen metabolism pathway: *CYP1B1* (c.1294C>G, rs1056836), *CYP3A4* (c.-392A>G, rs2740574), *CYP3A5* (c.219-237G>A, rs776746), *GSTM1/GSTT1* deletions, and *SULT1A1* (c.638G>A, rs9282861; and c.667A>G, rs1801030). Logistic regression was used to calculate the odds ratios (OR) with 95% confidence intervals (CIs), and unconditional logistic regression was used to estimate adjusted odds ratios (aORs) by ethnicity. Because of multiple testing, *p* values < 0.01 were significant after Bonferroni correction. *SULT1A1* (c.638G>A) was associated to infant acute lymphoblastic leukemia and acute myeloid leukemia (AML) risk in males (additive model: aOR = 0.52; 95% CI: 0.29–0.95, *p* = 0.03; dominant model: aOR = 2.18; 95% CI: 1.17–4.05, *p* = 0.01, respectively). *CYP1B1* polymorphism was associated with a decreased risk of AML either for non-white or female children (additive model: OR = 0.24; 95% CI: 0.08–0.76, *p* < 0.01; additive model: aOR = 0.27; 95% CI: 0.08–0.89, *p* = 0.03, respectively). Since polymorphisms of Cytochrome P450 genes presented gender-specific risk associations, we also investigated their expression. *CYP1B1* was not expressed in 57.1% of EAL cases, and its expression varied by genotype, gender, and leukemia subtype. Maternal-fetal *GSTT1* null genotype was associated with risk of EAL. This study shows that polymorphisms in genes of estrogen metabolism confer genetic susceptibility to EAL, mainly in males, and maternal susceptibility genes modify the risk for developing EAL in newborns.

## Introduction

The acute leukemia causation pathway has been postulated to be a multistep process comprising an interaction between environmental (e.g., ionizing radiation or carcinogenic substances such as benzene, tobacco, and estrogen) and genetic susceptibility factors [1]. Together, these factors may cause genetic or epigenetic alterations, destabilizing normal physiological conditions. In the leukemogenic pathway, a fetal hematopoietic stem cell acquires genetic alterations in utero, generating a pre-leukemic clone. The clonal proliferative advantage leads to the clinical onset of leukemia in early life [2].

Early age leukemia (EAL) has a short timeframe of exposure and the dose-effect in the causation pathways of EAL might be influenced by genetic susceptibility determined by multiple low-risk genetic variants. Most cases of EAL have somatic mutations, mainly rearrangements involving *MLL*, a master gene of hematopoiesis that regulates important genes of myeloid and lymphoid differentiation. Mutations arise in utero, as confirmed by backtracking methods using neonatal blood spots from children with acute leukemia [3]. *MLL* rearrangements (*MLL*-r) resemble those found in secondary acute myeloid leukemia (AML) resulting from exposure to topoisomerase II inhibitors, what makes it a model for leukemogenesis in EAL with *MLL*-r. Thus, it is hypothesized that inadvertent exposure to biochemically similar topoisomerase II inhibitors during pregnancy may be responsible for DNA breaks in fetal cells and the underlying mechanism of EAL [4, 5]. In order to test this hypothesis, we have previously demonstrated an association between maternal exposure to hormones (e.g., estrogen) before and during the first trimester of pregnancy and an increased risk of EAL. Hormonal exposure led to a more than 8-fold increased risk of developing infant leukemia [4]. The most pronounced risk was associated with hormonal consumption during the first trimester and among *MLL*-r cases (11-fold and 10-fold increased risk, respectively). The biological explanation for this observation was tested in a lymphoblastoid cell line, and a dose-dependent exposure to both estradiol and 4-hydroxiestradiol induced DNA double-strand breaks and *MLL*-r [6]. The rationale for the association between estrogen exposure and leukemogenesis in EAL was that estrogen metabolites lead to the formation of quinones and reactive oxygen species, promoting mutagenesis.

**Fig 1:**
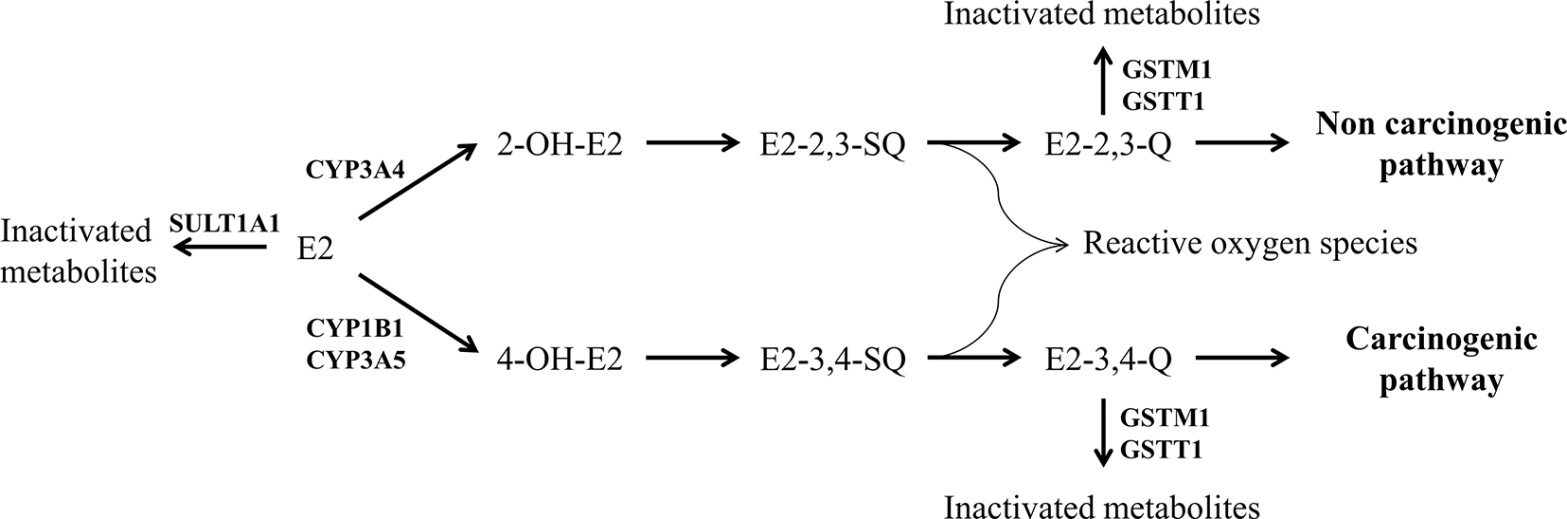
Metabolism of estrogen. Estradiol (E2) is converted to estrone and inactivated metabolites by SULT1A1. In addition, E2 hydrolization leads to either a carcinogenic or a non-carcinogenic pathway. 2-OH-E2, 2- hydroxyestradiol; 4-OH-E2, 4-hydroxyestradiol; E2-2,3-SQ, estradiol-2,3-semiquinone; E2-3,4-SQ, estradiol-3,4-semiquinone; E2-2,3-Q, estradiol-2,3-quinone; E2-3,4-Q, estradiol-3,4-quinone.

Important features of cancer susceptibility genes are their functional heterogeneity, as genetic polymorphisms might be associated to different enzyme activity/levels. Therefore, several susceptibility genes involved in the same metabolic pathway would influence the risk of developing EAL. It is important to highlight that susceptibility of cancer in early-life may be quite distinct from that observed in adulthood cancer development. Both estrogen metabolism (Fig 1) and xenobiotic metabolism are modulated by enzymes (i.e., CYPs, GSTs, and SULTs) that present genetic variants affecting their expression and/or activity. CYP1B1 catalyzes the biotransformation of estradiol to 4-hydroxiestradiol. A missense variant of *CYP1B1* (c.1294C>G, rs1056836) leads to a leucine to valine change at the protein level (p.L432V); the polymorphic allozyme exhibits greater activity toward estradiol hydroxylation than the wild-type [7]. Regarding the CYP3A family, CYP3A4 is responsible for 2-hydroxylation of estradiol in the liver, whereas CYP3A5 catalyzes 4-hydroxiestradiol. A polymorphism in the 5' promoter region of *CYP3A4* generates an adenine to guanine substitution (c.-392A>G, rs2740574), although the phenotypic consequence is controversy and unknown [8]. A polymorphism on *CYP3A5* íntron 3 (c.219-237G>A, rs776746) leads to the formation of an aberrant splice site and to the absence of the enzyme when a guanine is located at the polymorphic site [9]. Furthermore, the role of GSTs and SULTs in estrogen metabolism is related to theinactivation of estrogen and intermediate compounds, avoiding the formation of carcinogens such as 3,4-estradiolquinones. The human soluble GST family includes *GSTT1* and *GSTM1* at 22q11.2 and 1p13.3, respectively. Both genes can be completely deleted (null genotype), leading to the absence of these proteins [10]. Two polymorphisms in exon seven of *SULT1A1* have been reported to decrease both the activity and thermostability of the enzyme (c.638G>A, rs9282861 and c.667A>G, rs1801030) [11].

Since previous studies have reported an association between estrogen intake and leukemia, it is likely that estrogen metabolic pathway generates carcinogenic compounds and genomic alterations in a precursor clone. Estrogen metabolism comprises several enzymes coded by polymorphic genes, thus allowing a considerable metabolic range in the population. Based on the hypothesis that genetic polymorphisms of estrogen metabolism modulates the amount of carcinogenic compounds a precursor cell may be exposed, we evaluated five polymorphisms in *CYP1B1*, *CYP3A5*, *GSTM1*, *GSTT1*, and *SULT1A1* in order to investigate whether these genetic changes modify the risk of developing EAL.

## Materials and Methods

### Subjects

This case-control study included 754 children aged less than 24 months. Children were enrolled in the study from 2000 to 2012, and some cases and controls were included in previous reports that addressed questions related to maternal exposure during pregnancy [4, 12, 13]. Patients (*n* = 350) diagnosed with either acute lymphoblastic leukemia (ALL, *n* = 235) or AML (*n* = 115) were included. All controls (*n* = 404) were healthy and from the same Brazilian regions as the investigated cases. In addition, mothers of cases and controls who agreed to participate in this study by donating their biological material (*n* = 134) were genotyped.

Race/ethnicity was defined according to Instituto Brasileiro de Geografia e Estatística, which gathers subjects based on self-defined ethnicity. The subjects were categorized as white or non-white. The non-white group consisted of the participants who self-identified as brown or black (*www.ibge.gov.br*) [14].

The exclusion criteria for both cases and controls were as follows: syndromes (e.g., Down and myelodysplastic syndromes), T-cell precursor ALL, insufficient samples or bad DNA quality, and a lack of written consent from the mother.

Leukemia was diagnosed by immunomolecular analysis of the lymphoid and myeloid cells according to standard criteria as described previously [15].

### Ethical aspects

All collaborating Brazilian institutions approved the study, and written informed consent was obtained from the mother or relatives responsible for the enrolled children in accordance with the Declaration of Helsinki. The Ethics and Scientific Committees of Instituto Nacional de Câncer-INCA and the National Research Ethics Committee (CEP #005/06 and #024/10; CONEP #707/2010) approved this study.

### Genotyping

Genomic DNA was isolated from peripheral blood cells or buccal cells (controls, mothers). Among cases, DNA extraction was preferentially performed with samples lacking leukemic blast cells in order to rescue a maximum amount of constitutive DNA (e.g., remission samples, minimal residual disease). Isolation was carried out using either the QIAmp DNA Mini kit (Qiagen, Germany) or Oragene DNA technology (DNA Genotek Inc., Ontario, Canada) according to the manufacturers’ protocols.

The *CYP1B1* (c.1294C>G, rs1056836), *CYP3A4* (c.-392A>G, rs2740574), and *CYP3A5* (c.219-237G>A, rs776746) polymorphisms were genotyped by restriction fragment length polymorphism according to primer sequence designs described previously [16–18]. The polymerase chain reaction (PCR) products from *CYP1B1*, *CYP3A4*, and *CYP3A5* amplification were endonuclease-digested with the restriction enzymes *Ale*I, *Mbo*II, and *Ssp*I (Biolabs, England), respectively. The *GSTM1* and *GSTT1* homozygous deletion (null genotype) was detected with multiplex PCR in which an internal control (*CYP1A1*) was co-amplified [19]. The *SULT1A1* non-synonymous variants (c.638G>A, rs9282861 and c.667A>G, rs1801030) were genotyped by pyrosequencing on a PyroMark Q24 (Qiagen, Germany) after PCR amplification. Primers (forward, 5’-GTAATCCGAGCCTCCACTGA-3’; reverse, Biotin-5’-ACGACGTGTGCTGAACCA-3’; and sequencer, 5’-CCTGGAGTTTGTGGG-3’) flanked the region containing both polymorphisms. All conditions for the assays are available on request.

### Gene expression analysis

RNA was extracted from bone marrow samples using TRIzol reagent (Invitrogen, CA, USA) according to the manufacturer’s instructions. Following DNase treatment (Invitrogen, CA, USA), cDNA was synthesized using SuperScript III Reverse Transcriptase (Invitrogen, CA, USA) with 3 μg of purified RNA as a template. Real-time PCR was performed using Platinum SYBR Green qPCR Super Mix on a Rotor-Gene Q real time cycler (Qiagen, Germany). PCR was performed using diluted cDNA template in a 15 μL reaction mixture containing 0.30 μM of each primer and 7.5 μL of SYBR Green qPCR Mix. A melting curve phase program was applied with continuous fluorescence measurement between 60°C and 95°C. The negative controls for each primer consisted of a reaction with no cDNA added. The primer sequences for *CYP1B1*, *CYP3A4*, and *CYP3A5* are described elsewhere [20, 21].

The mRNA levels were normalized to glyceraldehyde-3-phosphate dehydrogenase expression using the following primers: 5’-CAACAGCCTCAAGATCATCAGCAA-3’ (forward) and 5’-AGTGATGGCATGGACTGTGGTCAT-3’ (reverse). Relative expression was determined using the 2^-ΔCt^ method [21]. The values are the medians of duplicates run for each analyzed sample.

### Statistical analysis

Sociodemographic variables (e.g., age at diagnosis, gender, and ethnicity) were compared between cases and controls using the Mann-Whitney and Pearson's chi-square (χ^2^) tests. The frequency of expected genetic polymorphism was calculated using the Hardy-Weinberg (HW) law based on the allele frequency in the control group. *p* Values < 0.05 were considered significant. The polymorphism frequency in children and mothers was compared to the control group using Chi-square (χ^2^) and Cochran Armitage Trend Tests. The disease risk was verified for the dominant, recessive and additive inheritance models; χ^2^ and Cochran Armitage Trend Test were used to calculate odds ratios (ORs) with 95% confidence intervals (CIs) and *p* values; unconditional logistic regression estimated adjusted odds ratios (aORs). Further Bonferroni’s correction for multiple testing was used; considering that five polymorphisms were analyzed, *p* values < 0.01 (0.05/5) indicated statistical significant results. Risk associations were calculated in the Statistical Product and Services Solutions statistical package, version 18.0 (SPSS Inc., Chicago, IL, USA), while Cochran Armitage Trend Test to evaluate inheritance models was analyzed with R software (http://www.r-project.org/). Statistical analyses concerning gene expression data were performed using GraphPad Prism 5 (GraphPad Software, Inc., California, USA) software. The comparison among genotypes in relation to *CYP1B1* and *CYP3A5* expression was performed using the Kruskal-Wallis and Dunn's Multiple Comparison tests. The Mann Whitney test was used for all other analyses.

## Results

The distribution of demographic and laboratory variables between cases (*n* = 350) and controls (*n* = 404) is provided in Table 1. All children were ≤ 2 years old, with a median age of 12 months at the time of the diagnosis of acute leukemia and 19 months at the identification of controls. The distribution of gender and ethnicity among cases and controls were similar (Table 1). The most frequent leukemia subtype was ALL (67.1%), and the rate of positivity for *MLL*-r was 45.9%.

**Table 1:** Descriptive table of cases and controls, Brazil, 2000–2012.

		Controls	Cases	
		(*n* = 404)	(*n* = 350)	
		*n* (%)	*n* (%)	*p *Value^a^
**Median age (months)**		19.0	12.0	
**Gender**				1.00
	**Male**	225 (55.7)	195 (55.7)	
	**Female**	179 (44.3)	155 (44.3)	
**Ethnicity**				0.25
	**White**	213 (57.4)	215 (61.6)	
	**Non-white**	158 (42.6)	134 (38.4)	
**Leukemia subtype**				
	**Infant ALL ^b^**	NA	124 (35.4)	
	**ALL^c^**	NA	111 (31.7)	
	**AML**	NA	115 (32.9)	
***MLL* status**				
	***MLL*-GL**	NA	164 (54.1)	
	***MLL-*r**	NA	139 (45.9)	

ALL, acute lymphoblastic leukemia; AML, acute myeloid leukemia; *MLL*-GL, *MLL* germ line; *MLL*-r, *MLL* rearranged; NA, not applicable

^a^ *p* Value corresponds to comparison of frequency distribution between controls and cases

^b^ infant ALL patients comprise children ≤ 12 months-old at diagnosis

^c^ ALL patients 13–24 months-old at diagnosis

For all controls, the allele frequencies for *CYP1B1* c.1294C>G, *CYP3A4* c.-392A>G, *CYP3A5* c.219-237G>A, *SULT1A1* c.638G>A, and *SULT1A1* c.667A>G were 0.50, 0.25, 0.30, 0.31, and 0.21, respectively. The *GSTM1* and *GSTT1* null genotypes were detected in 40.6% and 24.9% of controls, respectively. As shown in Table 2, the control genotypes for variant loci were in HW equilibrium, with the exception of the *CYP3A4* (c.-392A>G) and *SULT1A1* (c.638G>A) variants (*p* < 0.05). These deviations from HWE raise concern when genotypes are mistyped or a biased control population is included, however given that our population is known to be heterogeneous and it was equally stratified, we assume that the results reflect an intrinsic feature of this study population. Nevertheless, all results concerning *CYP3A4* c.-392A>G and *SULT1A1* c.638G>A are shown in the supporting information and were not reported as significant in this manuscript.

**Table 2:** Distribution of frequencies of variables analyzed in cases and controls, Brazil, 2000–2012.

		Controls (*n* = 404)			Cases (*n* = 350)
Genotypes		Total	Whites	Non-Whites	
		*n* (%)	*n* (%)	*n* (%)	*n* (%)
***CYP1B1* c.1294C>G**					
	**CC**	_78 (24.6)	45 (28.0)	25 (19.2)	_73 (28.4)
	**CG**	161 (50.8)	85 (52.8)	65 (50.0)	130 (50.6)
	**GG**	_78 (24.6)	31 (19.3)	40 (30.8)	_54 (21.0)
	***p* Value^a^**	0.78	0.42	0.88	NA
***CYP3A4* c.-392A>G**					
	**AA**	182 (59.1)	105 (63.6)	65 (53.3)	152 (58.0)
	**AG**	_95 (30.8)	45 (27.3)	41 (33.6)	_85 (32.4)
	**GG**	_31 (10.1)	15 (9.1)	16 (13.1)	_25 (_9.5)
	***p* Value^a^**	<0.01	<0.01	0.03	NA
***CYP3A5* c.219-237G>A**					
	**GG**	149 (50.7)	91 (55.5)	47 (44.8)	125 (48.8)
	**GA**	113 (38.4)	57 (34.8)	46 (43.8)	106 (41.4)
	**AA**	_32 (10.9)	16 (9.8)	12 (11.4)	_25 (_9.8)
	***p* Value^a^**	0.14	0.12	0.88	NA
***GSTM1***					
	**Non-null**	203 (59.4)	104 (57.1)	86 (65.4)	164 (56.6)
	**Null**	139 (40.6)	78 (42.9)	45 (34.4)	126 (43.4)
***GSTT1***					
	**Non-null**	257 (75.1)	147 (80.8)	93 (71.0)	215 (74.1)
	**Null**	_85 (24.9)	35 (19.2)	38 (29.0)	_75 (25.9)
***SULT1A1* c.638G>A**					
	**GG**	192 (47.5)	105 (50.5)	68 (43.3)	155 (48.0)
	**GA**	170 (42.1)	88 (42.3)	64 (40.8)	143 (44.3)
	**AA**	_42 (10.4)	15 (7.2)	25 (15.9)	_25 (_7.7)
	***p* Value^a^**	0.63	0.55	0.14	NA
***SULT1A1* c.667A>G**					
	**AA**	238 (58.9)	105 (50.5)	106 (67.5)	232 (71.8)
	**AG**	161 (39.9)	100 (48.1)	49 (31.2)	_87 (26.6)
	**GG**	5 (1.2)	3 (1.4)	2 (1.3)	5 (1.5)
	***p* Value^a^**	<0.01	<0.01	0.16	NA

NA, not applicable

^a^ *p* Value refers to analysis of HW equilibrium among controls

The genotype frequencies of *CYP1B1*, *CYP3A4*, *CYP3A5*, *GSTT1*, *GSTM1*, and *SULT1A1* according to ethnicity distribution among ALL (infant ALL, ALL 13–24 months) and AML are shown in S1 and S2 Tables. In whites, the *GSTT1* null genotype increased the risk of ALL in children 13–24 months-old (OR = 2.10; 95% CI, 1.04–4.25). The presence of the GG genotype of *CYP1B1* decreased the risk of developing AML (OR = 0.24; 95% CI, 0.08–0.76) in non-white children.

Because infants (≤ 12 months of age) have been relevant for ALL and associated with different B-cell precursor subtypes with *MLL-r*, only the ALL cases were sub-divided (infant ALL and ALL 13–24 months). The distributions of *CYP1B1*, *CYP3A4*, *CYP3A5*, *GSTT1*, *GSTM1*, and *SULT1A1* genotype frequencies according to leukemia subtype, with controls adjusted by ethnicity, are shown in S3 Table. Regarding ALL, a reduced the risk was associated to the AA genotype of *SULT1A1* (c.638G>A) only in infants (aOR = 0.24; 95% CI, 0.07–0.81), although the result was not statistically significant after Bonferroni correction (*p* Value = 0.02). Among AML cases, decreased risk was associated with the GG genotype of *CYP1B1* (aOR = 0.34; 95% CI, 0.16–0.75).

The risk associations were also tested in ALL and AML according to gender: males and females (Tables 3 and 4, respectively). In males, *SULT1A1* c.638G>A was negatively associated with infant ALL for the dominant (aOR = 0.52; 95% CI, 0.29–0.95) and additive models (aOR = 0.11; 95% CI, 0.01–0.83). On the other hand, the same polymorphism was associated with an increased risk of AML (dominant model: aOR = 2.18; 95% CI, 1.17–4.05) even after Bonferroni adjustment. In females, *CYP1B1* variant (additive model: aOR = 0.27; 95% CI, 0.08–0.89) was associated with decreased risk of AML.

**Table 3:** Genotype frequencies of *CYP1B1*, *CYP3A5*, *GSTT1*, *GSTM1* and *SULT1A1* in males and early age acute leukemia, Brazil, 2000–2012.

Genotypes/	Controls	iALL ^b^				ALL ^c^				AML			
Inheritance model		*n* (%)	aOR (95% CI) ^a^	*p* Value	*p* Value ^d^	*n* (%)	aOR (95% CI) ^a^	*p* Value	*p* Value ^d^	*n* (%)	aOR (95% CI) ^a^	*p* Value	*p* Value ^d^
***CYP1B1* c.1294C>G**													
**CC**	47 (27.2)	10 (20.8)		0.65	0.58	9 (18.8)		0.43	0.21	18 (39.1)		0.20	0.07
**CG**	83 (48.0)	26 (54.2)				24 (50.0)				21 (45.7)			
**GG**	43 (24.9)	12 (25.0)				15 (31.2)				7 (15.2)			
**Dominant model**			1.54 (0.70–3.36)	0.28	0.37		1.67 (0.74–3.77)	0.21	0.24		0.60 (0.30–1.21)	0.15	0.11
**Recessive model**			1.10 (0.52–2.35)	0.80	0.98		1.38 (0.67–2.86)	0.38	0.37		0.49 (0.19–1.25)	0.14	0.17
**Additive model**			1.40 (0.54–3.65)	0.49	0.57		1.82 (0.69–4.84)	0.23	0.20		0.42 (0.15–1.19)	0.10	0.08
***CYP3A5* c.219-237G>A**													
**GG**	92 (54.8)	30 (57.7)		0.91	0.67	26 (49.1)		0.41	0.85	17 (42.5)		**0.03**	0.77
**GA**	57 (33.9)	17 (32.7)				23 (43.4)				22 (55.0)			
**AA**	19 (11.3)	5 (9.6)				4 (7.5)				1 (2.5)			
**Dominant model**			0.90 (0.48–1.70)	0.75	0.71		1.27 (0.68–2.37)	0.46	0.47		1.59 (0.79–3.24)	0.20	0.16
**Recessive model**			0.91 (0.32–2.63)	0.87	0.73		0.71 (0.23–2.22)	0.55	0.43		0.23 (0.03–1.75)	0.15	0.09
**Additive model**			0.88 (0.30–2.61)	0.82	0.69		0.82 (0.25–2.67)	0.74	0.62		0.31 (0.04–2.46)	0.27	0.21
***GSTM1***													
**Non-null**	121 (64.4)	35 (61.4)	1.00			30 (55.6)	1.00			29 (52.7)	1.00		
**Null**	67 (35.6)	22 (38.6)	1.20 (0.64–2.24)	0.57	0.68	24 (44.4)	1.61 (0.86–3.00)	0.14	0.24	26 (47.3)	1.82 (0.98–3.38)	0.06	0.11
***GSTT1***													
**Non-null**	136 (72.3)	39 (68.4)	1.00			37 (68.5)	1.00			43 (78.2)	1.00		
**Null**	52 (27.7)	18 (31.6)	1.28 (0.67–2.48)	0.46	0.57	17 (31.5)	1.23 (0.63–2.39)	0.54	0.58	12 (21.8)	0.70 (0.33–1.48)	0.35	0.39
***SULT1A1* c.638G>A**													
**GG**	105 (47.3)	39 (63.9)		0.20	**0.006**	32 (57.1)		0.29	0.12	19 (31.1)		0.08	**0.04**
**GA**	93 (41.9)	21 (34.4)				21 (37.5)				33 (54.1)			
**AA**	24 (10.8)	1 (1.6)				3 (5.4)				9 (14.8)			
**Dominant model**			**0.52 (0.29–0.95)**	**0.03**	**0.02**		0.68 (0.37–1.23)	0.20	0.19		**2.18 (1.17–4.05)**	**<0.01** *	**0.02**
**Recessive model**			0.14 (0.02–1.03)	0.05	**0.03**		0.40 (0.12–1.39)	0.15	0.22		1.40 (0.60–3.23)	0.44	0.40
**Additive model**			**0.11 (0.01–0.83)**	**0.03**	**<0.01** *		0.37 (0.10–1.33)	0.13	0.16		2.18 (0.85–5.56)	0.11	0.11

ALL, acute lymphoblastic leukemia; AML, acute myeloid leukemia; aOR, adjusted odds ratio; CI, confidence intervals; iALL, infant ALL.

^a^ Odds ratio adjusted by skin color.

^b^ infant ALL patients comprise children ≤ 12 months-old at diagnosis.

^c^ ALL patients 13–24 months-old at diagnosis.

^d^ Cochran Armitage Test.

* Statistically significant (*p* Value < 0.01) after Bonferroni correction.

**Table 4:** Genotype frequencies of *CYP1B1*, *CYP3A5*, *GSTT1*, *GSTM1* and *SULT1A1* in females and early age acute leukemia, Brazil, 2000–2012.

Genotypes/	Controls	iALL ^b^				ALL ^c^				AML			
Inheritance model		*n* (%)	aOR (95% CI) ^a^	*p* Value	*p* Value ^d^	*n* (%)	aOR (95% CI) ^a^	*p* Value	*p* Value ^d^	*n* (%)	aOR (95% CI) ^a^	*p* Value	*p* Value ^d^
***CYP1B1* c.1294C>G**													
**CC**	30 (21.4)	12 (29.3)		0.36	0.16	10 (29.4)		0.48	0.70	13 (33.3)		0.62	0.06
**CG**	77 (55.0)	23 (56.1)				15 (44.1)				21 (53.8)			
**GG**	33 (23.6)	6 (14.6)				9 (26.5)				5 (12.8)			
**Dominant model**			0.57 (0.25–1.28)	0.17	0.30		0.58 (0.24–1.36)	0.21	0.32		0.47 (0.21–1.04)	0.06	0.12
**Recessive model**			0.56 (0.21–1.45)	0.23	0.22		1.13 (0.48–2.66)	0.79	0.72		0.44 (0.16–1.22)	0.11	0.15
**Additive model**			0.40 (0.13–1.22)	0.11	0.15		0.75 (0.26–2.17)	0.60	0.70		**0.27 (0.08–0.89)**	**0.03**	0.07
***CYP3A5* c.219-237G>A**													
**GG**	55 (44.7)	19 (46.3)		0.71	0.84	15 (48.4)		0.28	0.71	18 (46.2)		0.72	0.72
**GA**	55 (44.7)	16 (39.0)				10 (32.3)				18 (46.2)			
**AA**	13 (10.6)	6 (14.6)				6 (19.4)				3 (7.7)			
**Dominant model**			0.98 (0.47–2.03)	0.96	0.86		0.94 (0.42–2.12)	0.88	0.71		0.80 (0.37–1.75)	0.58	0.87
**Recessive model**			1.47 (0.51–4.22)	0.48	0.48		2.11 (0.72–6.20)	0.18	0.18		1.24 (0.35–4.36)	0.60	0.60
**Additive model**			1.25 (0.40–3.90)	0.70	0.60		1.68 (0.53–5.34)	0.38	0.36		0.52 (0.12–2.24)	0.38	0.61
***GSTM1***													
**Non-null**	81 (54.4)	28 (63.6)	1.00			16 (48.5)	1.00			26 (55.3)	1.00		
**Null**	68 (45.6)	16 (36.4)	0.64 (0.32–1.29)	0.21	0.28	17 (51.5)	1.22 (0.57–2.61)	0.61	0.54	21 (44.7)	0.95 (0.49–1.84)	0.87	0.91
***GSTT1***													
**Non-null**	119 (79.9)	34 (77.3)	1.00			28 (84.8)	1.00			34 (72.3)	1.00		
**Null**	30 (20.1)	10 (22.7)	1.32 (0.58–3.01)	0.51	0.71	5 (15.2)	0.79 (0.28–2.25)	0.66	0.51	13 (27.7)	1.60 (0.73–3.51)	0.24	0.30
***SULT1A1* c.638G>A**													
**GG**	83 (47.2)	19 (38.8)		0.14	0.83	19 (40.4)		0.69	0.40	27 (55.1)		0.35	0.35
**GA**	75 (42.6)	28 (57.1)				22 (46.8)				18 (36.7)			
**AA**	18 (10.2)	2 (4.1)				6 (12.8)				4 (8.2)			
**Dominant model**			1.43 (0.74–2.75)	0.29	0.30		1.32 (0.68–2.56)	0.41	0.41		0.72 (0.38–1.36)	0.31	0.33
**Recessive model**			0.41 (0.09–1.85)	0.25	0.18		1.41 (0.52–3.86)	0.50	0.62		0.79 (0.25–2.51)	0.69	0.67
**Additive model**			0.54 (0.11–2.57)	0.44	0.35		1.58 (0.54–4.60)	0.41	0.48		0.67 (0.21–2.21)	0.52	0.52

ALL, acute lymphoblastic leukemia; AML, acute myeloid leukemia; aOR, adjusted odds ratio; CI, confidence intervals; iALL, infant ALL.

^a^ Odds ratio adjusted by skin color.

^b^ infant ALL patients comprise children ≤ 12 months-old at diagnosis.

^c^ ALL patients 13–24 months-old at diagnosis.

^d^ Cochran Armitage Test.

Another aim of this study was to focus on the sum of low penetrance variants of the xenobiotic system (phase I and phase II) that can enhance the risk of EAL. The combinations of *CYP1B1*, *CYP3A4*, *CYP3A5*, *GSTT1*, *GSTM1*, and *SULT1A1* genotypes are shown in S6 Table. None of the combinations between *CYP1B1/CYP3A5* and *GSTT1*/GSTM1/SULT1A1*2 retrieved significant results. The associations of genetic susceptibility with EAL risk stratified according to *MLL* status were also tested, but only the heterozygous genotype of *CYP3A5* presented a significant increase of AML risk in *MLL*-GL cases (aOR = 2.21; 95% CI, 1.12–4.35), though the significance did not remain after multiple testing adjustment (S7 Table).

Considering that differences in susceptibility, mainly during the fetal period, are dependent on the maternal system and placental metabolism, maternal genotypes were also tested, as shown in S8 Table. The *SULT1A1* (c.638G>A) AA genotype reduced the risk of EAL (aOR = 0.16; 95% CI, 0.03–0.78). The combined mother/child genotype was assessed to evaluate the association with EAL risk (S9 Table). The *GSTT1* null genotype in both mother and child was associated with an increased risk of EAL in newborns (aOR = 15.0; 95% CI, 1.22–185.0). However, both results lost their significance with Bonferroni correction.

Because *CYP1B1* polymorphism (c.1294C>G) was associated with a decreased risk of AML, which contradicts the initial hypothesis, we tested whether the genotypes of *CYP1B1*, *CYP3A4*, and *CYP3A5* correlate with their own gene expression (S10 Table). We have analyzed mainly bone marrow aspirates in order to confirm the gene expression in hematopoietic cells. *CYP1B1* expression was found in a greater proportion of *CYP1B1* wild-type genotype patients (44%) than CG and GG genotype carriers (21%; *P* = 0.04). Accordingly, the majority of the CG and GG genotype carriers with ALL (85%) or AML (67%) did not express the gene. No gene expression was found in any of the patients tested for *CYP3A4* (*n* = 40). Also, no significant correlation was observed between *CYP3A5* genotypes and gene expression.

**Fig 2:**
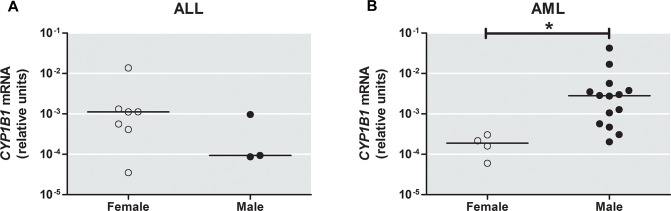
Differential levels of *Cytochrome P450 1B1* (*CYP1B1*) expression according to gender. (A) Comparison of *CYP1B1* expression between females and males with acute lymphoblastic leukemia, ALL. (B) *CYP1B1* mRNA levels was higher in males than females with acute myeloid leukemia, AML. Horizontal bars indicate median value; * Significant *p* Value (*P* = 0.007).

Additionally we tested whether *CYP1B1*, *CYP3A4*, and *CYP3A5* expression varied according to gender. *CYP1B1* expression was detected in 29% of females and 30% of males, whereas *CYP3A5* expression was detected in 14% of females and 16% of males. No association was found between gender and *CYP3A5* expression. Regarding *CYP1B1* and leukemia subtype, higher levels of gene expression were detected in girls with ALL (Fig 2A) and in boys with AML (Fig 2B).

## Discussion

Here, we assessed the associations of genetic susceptibility in the natural history of EAL. Our hypothesis was based on previous epidemiological data suggesting that maternal exposure to estradiol during the prenatal period is associated with an increased risk of EAL [4]. As constitutive gene variants modify the dose effect of a particular exposure—a paradigm in carcinogenesis—the rationale for this study was to evaluate possible alterations in genes coding for key enzymes in phase I and II of xenobiotic metabolism. The heterogeneity of Brazilian population may explain the significant differences of some allele frequencies observed and, aware of this, all the analyses were adjusted by ethnicity. Certainly, the risk associations should be interpreted carefully with regard to statistical calculations. Even so, these differences represent an important study strength, which is to exemplify how the admixture of Brazilians has an important impact on many aspects of medicine genetics. As shown by Pena *et al*., the conventional socio-demographic categories are not able to represent the heterogeneity of our population [14]; therefore, in the context of molecular population genetics, this is a great contribution of this study because only relying on data from more homogeneous populations would result in erroneous assertions.

A hurdle in EAL is to understand the pathways that lead leukemogenesis toward the lymphoblastic or myeloid phenotype. For example, age-specific subtypes are well-defined in ALL cases, whereas the influence of age in AML cases is less remarkable, possibly reflecting different etiologies. Therefore, we analyzed the genetic polymorphisms by stratifying the cases according to diagnosis (ALL vs. AML). Overall, our data show that the *CYP1B1* (phase I metabolism) genetic polymorphism significantly modifies the risk in AML patients, whereas *SULT1A1* polymorphisms (phase II metabolism) modify the risk in ALL patients.

The G allele of *CYP1B1* (c.1294C>G) presents a gain of activity compared to the wild-type isoform [7, 22], therefore, we speculated that the variant allele of *CYP1B1* may increase EAL risk by inducing the formation of quinones and reactive oxygen species; however, our results show that the GG genotype is associated with a decreased risk of AML. Helmig *et al*. demonstrated that *CYP1B1* polymorphic homozygotes have lower mRNA expression [23]. In addition, carriers of the C allele have a greater response to environmental factors, whereas GG homozygotes do not exhibit increased expression after xenobiotic influence [24]. Thus, lower *CYP1B1* expression could compensate for the higher protein activity, possibly explaining the decreased risk observed in the present study. Our observations were in accordance with Helmig *et al*.’s study, as approximately 80% of children with CG/GG genotypes did not express the gene. Remarkably, the immaturity of the xenobiotic-metabolizing system was verified in this study, as the majority of the children did not express *CYP1B1*, *CYP3A4*, and *CYP3A5*.

Several studies have investigated the association of *SULT1A1* variants with cancer, and the results have been controversial [25, 26]. In the current study, *SULT1A1* (c.638G>A) was associated with a reduced risk of ALL. This decreased risk of ALL is not compatible with our hypothesis. Estrogen and its derivatives, which stimulate proliferation, are deactivated by sulfation because this conjugating reaction yields estrone-sulfate, a circulating form of estrogen that may be excreted [27]. Because the *SULT1A1* polymorphism decreases enzymatic activity and thermostability, *SULT1A1* polymorphism may increase the bioavailability of estrogens, leading to the formation of DNA adducts [11, 28]. Notably, the greater a person’s age, the greater the SULT1A1 activity in the liver [27]; therefore, the infants may be more susceptible to the impact of the *SULT1A1* polymorphism.

EAL is more frequent in males than females, but the reasons for this discrepancy are not yet understood. We observed some genetic polymorphism associations related to gender. An association of *SULT1A1* (c.638G>A) in males and acute leukemia was found. Studies performed in animal models have revealed that female mice express higher levels of *SULT1A1* than male mice [29, 30]. The difference was attributed to the inhibitory effects of growth hormone and androgens in males. Gonadectomy in males resulted in mRNA levels similar to those observed in females [29]. We hypothesize that sexual differences in *SULT1A1* expression and its polymorphic variant may influence the metabolism of compounds associated with the initiation of EAL.

The influence of maternal metabolism of estrogens and other compounds on fetal exposure levels is important. EAL is known to be initiated in utero and the primary interface between maternal and fetal circulation is the placenta, and the placental micro-environment is thought to be very important for acute leukemia predisposition. Many of the environmental exposures with a capacity to alter fetal development are likely to be modulated by maternal and fetal genetic factors. The presence of the *SULT1A1* (c.638G>A) homozygous genotype in mothers was associated with a decreased risk of EAL. Because sulfated hormones serve as circulating and stock forms, the reduced activity of the polymorphic SULT1A1 may decrease the bioavailability of estrogen to the fetal circulation thus protecting it from DNA damage [27]. The *GSTT1* null genotype in both mother and child was associated with an increased risk of EAL. A recent study found that maternal haplotypes of *GSTM3*/*GSTM4* and *GSTP1* increase the risk of childhood ALL [31]. Future exploration of combined environmental and genetic approaches is likely to unravel the mechanisms underlying the gene-environment interaction at the fetal-maternal interface.

This study has some limitations. First, the sample size is impacted by the heterogeneity of leukemia subtypes (infant ALL, AML and ALL) that could have distinct pathogenesis. Second, maternal samples were not available for every case and control, and exposure data was restricted to a small number of cases and controls (11.4% and 15.2%, respectively) that had biological material available for genotyping. It limited the analysis of an interaction between estrogen, other xenobiotic exposures and genetic polymorphism data. However, this study also has some strengths. First, a great number of infant leukemia, an extremely rare disease, was included; these numbers allowed us, for example, to find differences regarding gender and to evaluate genotype combinations. Second, the hypothesis is well supported by previous observational and experimental data on the association between estrogen exposure and leukemia development. Third, this report is the first to evaluate the association between genetic variability in *SULT1A1* and susceptibility to childhood leukemia. Fourth, gene expression results were used as complementary data for understanding the associations between genetic polymorphisms and EAL. Finally, the enrollment of children from different Brazilian regions enables broader interpretation of data than studies performed in single cities and/or institutions.

In conclusion, *CYP1B1*, *CYP3A5*, *GSTT1* and *SULT1A1* variants exhibit significant risk associations with EAL. Gender is an important modulator of risk and may explain certain aspects related to the male/female incidence of EAL. Furthermore, genetic polymorphisms of mothers and their combination with child genotype supports the initial hypothesis and reveals that therisk association between EAL and polymorphisms of phase I and II estrogen metabolism are also dependent on both mother and child genetic susceptibility factors.

## Supporting Information

S1 Table Genotype frequencies of *CYP1B1*, *CYP3A4*, *CYP3A5*, *GSTT1*, *GSTM1* and *SULT1A1* according to white skin color in early age acute leukemia, Brazil, 2000–2012. (DOC)

S2 Table Genotype frequencies of *CYP1B1*, *CYP3A4*, *CYP3A5*, *GSTT1*, *GSTM1* and *SULT1A1* according to non-white skin color in early age acute leukemia, Brazil, 2000–2012. (DOC)

S3 Table Genotype frequencies of *CYP1B1*, *CYP3A4*, *CYP3A5*, *GSTT1*, *GSTM1* and *SULT1A1* according to age at diagnosis and leukemia subtypes, Brazil, 2000–2012. (DOC)

S4 Table Genotype frequencies of *CYP3A4* and *SULT1A1* in males and early age acute leukemia, Brazil, 2000–2012. (DOC)

S5 Table Genotype frequencies of *CYP3A4* and *SULT1A1* in females and early age acute leukemia, Brazil, 2000–2012. (DOC)

S6 Table Combination of genotype frequencies of *CYP1B1*, *CYP3A4*, *CYP3A5*, *GSTT1*, *GSTM1* and *SULT1A1* and leukemia subtypes, Brazil, 2000–2012. (DOC)

S7 Table The genotype risk association in acute leukemia with different *MLL* status, Brazil, 2000–2012. (DOC)

S8 Table Genotype frequencies of *CYP1B1*, *CYP3A4*, *CYP3A5*, *GSTT1*, *GSTM1* and *SULT1A1* in mothers of cases and controls, Brazil, 2000–2012. (DOC)

S9 Table Mother-child paired genotypes, Brazil, 2000–2012. (DOC)

S10 Table Gene expression according to child genotype, Brazil, 2000–2012. (DOC)
