# Supplementary material for: Polymorphisms in CYP1B1, CYP3A5, GSTT1, and SULT1A1 Are Associated with Early Age Acute Leukemia
Source: PLoS One. 2015 May 18;10(5):e0127308. doi: 10.1371/journal.pone.0127308 (PMC4436276; doi:10.1371/journal.pone.0127308)
Supplement: S1 Table — (DOC) [file pone.0127308.s001.doc]

**S1 Table. Genotype frequencies of *CYP1B1*, *CYP3A4*, *CYP3A5*, *GSTT1, GSTM1* and *SULT1A1* according to white skin color in early age acute leukemia, Brazil, 2000-2012.**

| **Genotypes** | **Controls** | **iALL^a^** | **OR (95% CI)** | ***p* Value** | **ALL^b^** | **OR (95% CI)** | ***p* Value** | **AML** | **OR (95% CI)** | ***p* Value** |
| --- | --- | --- | --- | --- | --- | --- | --- | --- | --- | --- |
| ***CYP1B1* c.1294C>G** |  |  |  |  |  |  |  |  |  |  |
| **CC** | 45 (28.0) | 12 (20.3) | 1.00 |  | 14 (30.4) | 1.00 |  | 18 (38.3) | 1.00 |  |
| **CG** | 85 (52.8) | 36 (61.0) | 1.59 (0.75–3.35) | 0.22 | 21 (45.7) | 0.79 (0.37–1.71) | 0.56 | 23 (48.9) | 0.68 (0.33–1.38) | 0.28 |
| **GG** | 31 (19.3) | 11 (18.6) | 1.33 (0.52–3.40) | 0.55 | 11 (23.9) | 1.14 (0.46–2.84) | 0.78 | 6 (12.8) | 0.48 (0.17–1.36) | 0.16 |
| ***CYP3A4* c.-392A>G** |  |  |  |  |  |  |  |  |  |  |
| **AA** | 105 (63.6) | 38 (64.4) | 1.00 |  | 31 (70.5) | 1.00 |  | 35 (66.0) | 1.00 |  |
| **AG** | 45 (27.3) | 15 (25.4) | 0.92 (0.46–1.84) | 0.82 | 11 (25.0) | 0.83 (0.38–1.79) | 0.63 | 16 (30.2) | 1.07 (0.54–2.12) | 0.85 |
| **GG** | 15 (9.1) | 6 (10.2) | 1.11 (0.40–3.06) | 0.85 | 2 (4.5) | 0.45 (0.10–2.08) | 0.37 | 2 (3.8) | 0.40 (0.09–1.84) | 0.23 |
| ***CYP3A5* c.219-237G>A** |  |  |  |  |  |  |  |  |  |  |
| **GG** | 91 (55.5) | 30 (50.8) | 1.00 |  | 28 (56.0) | 1.00 |  | 23 (53.5) | 1.00 |  |
| **GA** | 57 (34.8) | 22 (37.3) | 1.17 (0.62–2.23) | 0.63 | 16 (32.0) | 0.91 (0.45–1.83) | 0.80 | 19 (44.2) | 1.32 (0.66–2.64) | 0.43 |
| **AA** | 16 (9.8) | 7 (11.9) | 1.33 (0.50–3.53) | 0.57 | 6 (12.0) | 1.22 (0.44–3.41) | 0.71 | 1 (2.3) | 0.25 (0.03–1.96) | 0.20 |
| ***GSTM1*** |  |  |  |  |  |  |  |  |  |  |
| **Non-null** | 104 (57.1) | 39 (58.2) | 1.00 |  | 26 (54.2) | 1.00 |  | 33 (55.9) | 1.00 |  |
| **Null** | 78 (42.9) | 28 (41.8) | 0.96 (0.54–1.69) | 0.88 | 22 (45.8) | 1.13 (0.60–2.14) | 0.71 | 26 (44.1) | 1.05 (0.58–1.90) | 0.87 |
| ***GSTT1*** |  |  |  |  |  |  |  |  |  |  |
| **Non-null** | 147 (80.8) | 47 (70.1) | 1.00 |  | 32 (66.7) | 1.00 |  | 48 (81.4) | 1.00 |  |
| **Null** | 35 (19.2) | 20 (29.9) | 1.79 (0.94–3..39) | 0.07 | 16 (33.3) | **2.10 (1.04–4.25)** | **0.04** | 11 (18.6) | 0.96 (0.45–2.04) | 0.92 |
| ***SULT1A1* c.638G>A** |  |  |  |  |  |  |  |  |  |  |
| **GG** | 105 (50.5) | 38 (50.7) | 1.00 |  | 30 (49.2) | 1.00 |  | 23 (36.5) | 1.00 |  |
| **GA** | 88 (42.3) | 34 (45.3) | 1.07 (0.62–1.84) | 0.81 | 25 (41.0) | 0.99 (0.55–1.82) | 0.99 | 32 (50.8) | 1.66 (0.91–3.04) | 0.10 |
| **AA** | 15 (7.2) | 3 (4.0) | 0.55 (0.15–2.02) | 0.57 | 6 (9.8) | 1.40 (0.50–3.92) | 0.58 | 8 (12.7) | 2.44 (0.92–6.42) | 0.07 |
| ***SULT1A1* c.667A>G** |  |  |  |  |  |  |  |  |  |  |
| **AA** | 105 (50.5) | 52 (69.3) | 1.00 |  | 49 (80.3) | 1.00 |  | 54 (85.7) | 1.00 |  |
| **AG** | 100 (48.1) | 23 (30.7) | 0.46 (0.27–0.82) | 0.007* | 12 (19.7) | 0.26 (0.13–0.51) | <0.001* | 8 (12.7) | 0.16 (0.07–0.34) | < 0.001* |
| **GG** | 3 (1.4) | 0 (0.0) |  |  | 0 (0.0) |  |  | 1 (1.6) | 0.65 (0.07–6.38) | 1.00 |

ALL, acute lymphoblastic leukemia; AML, acute myeloid leukemia; CI, confidence intervals; iALL, infant ALL; OR, odds ratio.

^a^ infant ALL patients comprise children ≤ 12 months-old at diagnosis.

^b^ ALL patients 13-24 months-old at diagnosis.

* Statistically significant (p Value < 0.01) after Bonferroni correction.
