# Supplementary material for: Polymorphisms in CYP1B1, CYP3A5, GSTT1, and SULT1A1 Are Associated with Early Age Acute Leukemia
Source: PLoS One. 2015 May 18;10(5):e0127308. doi: 10.1371/journal.pone.0127308 (PMC4436276; doi:10.1371/journal.pone.0127308)
Supplement: S2 Table — (DOC) [file pone.0127308.s002.doc]

**S2 Table. Genotype frequencies of *CYP1B1*, *CYP3A4*, *CYP3A5*, *GSTT1, GSTM1* and *SULT1A1* according to non-white skin color in early age acute leukemia, Brazil, 2000-2012.**

| **Genotypes** | **Controls** | **iALL^a^** | **OR (95% CI)** | ***p* Value** | **ALL^b^** | **OR (95% CI)** | ***p* Value** | **AML** | **OR (95% CI)** | ***p* Value** |
| --- | --- | --- | --- | --- | --- | --- | --- | --- | --- | --- |
| ***CYP1B1* c.1294C>G** |  |  |  |  |  |  |  |  |  |  |
| **CC** | 25 (19.2) | 10 (33.3) | 1.00 |  | 5 (13.9) | 1.00 |  | 13 (35.1) | 1.00 |  |
| **CG** | 65 (50.0) | 13 (43.3) | 0.50 (0.19–1.29) | 0.15 | 18 (50.0) | 1.39 (0.46–4.13) | 0.56 | 19 (51.4) | 0.56 (0.24–1.31) | 0.18 |
| **GG** | 40 (30.8) | 7 (23.3) | 0.44 (0.15–1.30) | 0.13 | 13 (36.1) | 1.63 (0.52–5.11) | 0.40 | 5 (13.5) | **0.24 (0.08–0.76)** | **<0.01*** |
| ***CYP3A4* c.-392A>G** |  |  |  |  |  |  |  |  |  |  |
| **AA** | 65 (53.3) | 18 (58.1) | 1.00 |  | 17 (45.9) | 1.00 |  | 13 (35.1) | 1.00 |  |
| **AG** | 41 (33.6) | 8 (25.8) | 0.71 (0.28–1.77) | 0.45 | 14 (37.8) | 1.31 (0.58–2.93) | 0.52 | 20 (54.1) | 2.44 (1.10–5.43) | 0.03 |
| **GG** | 16 (13.1) | 5 (16.1) | 1.13 (0.36–3.50) | 0.78 | 6 (16.2) | 1.43 (0.49–4.22) | 0.57 | 4 (10.8) | 1.25 (0.36–4.35) | 0.75 |
| ***CYP3A5* c.219-237G>A** |  |  |  |  |  |  |  |  |  |  |
| **GG** | 47 (44.8) | 19 (55.9) | 1.00 |  | 13 (38.2) | 1.00 |  | 12 (34.3) | 1.00 |  |
| **GA** | 46 (43.8) | 11 (32.4) | 0.59 (0.25–1.38) | 0.22 | 17 (50.0) | 1.34 (0.58–3.06) | 0.49 | 20 (57.1) | 1.70 (0.75–3.88) | 0.20 |
| **AA** | 12 (11.4) | 4 (11.8) | 0.83 (0.24–2.88) | 1.00 | 4 (11.8) | 1.21 (0.33–4.37) | 0.78 | 3 (8.6) | 0.98 (0.24–4.03) | 1.00 |
| ***GSTM1*** |  |  |  |  |  |  |  |  |  |  |
| **Non-null** | 86 (65.4) | 24 (70.6) | 1.00 |  | 20 (51.3) | 1.00 |  | 21 (50.0) | 1.00 |  |
| **Null** | 45 (34.4) | 10 (29.4) | 0.80 (0.35–1.81) | 0.59 | 19 (48.7) | 1.82 (0.88–3.75) | 0.10 | 21 (50.0) | 1.91 (0.95–3.87) | 0.07 |
| ***GSTT1*** |  |  |  |  |  |  |  |  |  |  |
| **Non-null** | 93 (71.0) | 26 (76.5) | 1.00 |  | 33 (84.6) | 1.00 |  | 29 (69.0) | 1.00 |  |
| **Null** | 38 (29.0) | 8 (23.5) | 0.75 (0.31–1.81) | 0.53 | 6 (15.4) | 0.45 (0.17–1.15) | 0.09 | 13 (31.0) | 1.10 (0.52–2.34) | 0.81 |
| ***SULT1A1* c.638G>A** |  |  |  |  |  |  |  |  |  |  |
| **GG** | 68 (43.3) | 20 (57.1) | 1.00 |  | 21 (50.0) | 1.00 |  | 22 (47.8) | 1.00 |  |
| **GA** | 64 (40.8) | 15 (42.9) | 0.80 (0.38–1.69) | 0.55 | 18 (42.9) | 0.91 (0.45–1.86) | 0.80 | 19 (41.3) | 0.92 (0.46–1.85) | 0.81 |
| **AA** | 25 (15.9) | 0 (0.0) |  |  | 3 (7.1) | 0.39 (0.11–1.42) | 0.14 | 5 (10.9) | 0.62 (0.21–1.81) | 0.38 |
| ***SULT1A1* c.667A>G** |  |  |  |  |  |  |  |  |  |  |
| **AA** | 106 (67.5) | 18 (51.4) | 1.00 |  | 28 (66.7) | 1.00 |  | 30 (65.2) | 1.00 |  |
| **AG** | 49 (31.2) | 16 (45.7) | 1.92 (0.91–4.09) | 0.09 | 11 (26.2) | 0.85 (0.39–1.85) | 0.68 | 16 (34.8) | 1.15 (0.58–2.31) | 0.69 |
| **GG** | 2 (1.3) | 1 (2.9) | 2.94 (0.25–34.2) | 0.39 | 3 (7.1) | 5.68 (0.91–35.7) | 0.07 | 0 (0.0) |  |  |

ALL, acute lymphoblastic leukemia; AML, acute myeloid leukemia; CI, confidence intervals; iALL, infant ALL; OR, odds ratio.

^a^ infant ALL patients comprise children ≤ 12 months-old at diagnosis.

^b^ ALL patients 13-24 months-old at diagnosis.

* Statistically significant (p Value < 0.01) after Bonferroni correction.
