# Supplementary material for: Polymorphisms in CYP1B1, CYP3A5, GSTT1, and SULT1A1 Are Associated with Early Age Acute Leukemia
Source: PLoS One. 2015 May 18;10(5):e0127308. doi: 10.1371/journal.pone.0127308 (PMC4436276; doi:10.1371/journal.pone.0127308)
Supplement: S3 Table — (DOC) [file pone.0127308.s003.doc]

**S3 Table. Genotype frequencies of *CYP1B1*, *CYP3A4*, *CYP3A5*, *GSTT1,* *GSTM1* and *SULT1A1* according to age at diagnosis and leukemia subtypes, Brazil, 2000-2012.**

| **Genotypes** | **Controls** | **Overall Cases** | | | **Infant ALL** | | | **ALL 13-24 months** | | | **AML^a^** | | |
| --- | --- | --- | --- | --- | --- | --- | --- | --- | --- | --- | --- | --- | --- |
|  |  | ***n* (%)** | **aOR (95% CI) ^b^** | ***p* Value** | ***n* (%)** | **aOR (95% CI) ^b^** | ***p* Value** | ***n* (%)** | **aOR (95% CI) ^b^** | ***p* Value** | ***n* (%)** | **aOR (95% CI) ^b^** | ***p* Value** |
| ***CYP1B1* c.1294C>G** |  |  |  |  |  |  |  |  |  |  |  |  |  |
| **CC** | 78 (24.6) | 72 (28.1) | 1.00 |  | 22 (24.7) | 1.00 |  | 19 (23.2) | 1.00 |  | 31 (36.5) | 1.00 |  |
| **CG** | 161 (50.8) | 130 (50.8) | 0.85 (0.56–1.27) | 0.42 | 49 (55.1) | 1.04 (0.59–1.86) | 0.88 | 39 (47.6) | 0.96 (0.52–1.79) | 0.90 | 42 (49.4) | 0.63 (0.36–1.08) | 0.09 |
| **GG** | 78 (24.6) | 54 (21.1) | 0.74 (0.45–1.20) | 0.22 | 18 (20.2) | 0.82 (0.40–1.67) | 0.59 | 24 (29.3) | 1.31 (0.65–2.65) | 0.45 | 12 (14.1) | **0.34 (0.16–0.75)** | **< 0.01*** |
| ***CYP3A4* c.-392A>G** |  |  |  |  |  |  |  |  |  |  |  |  |  |
| **AA** | 182 (59.1) | 152 (58.0) | 1.00 |  | 56 (62.2) | 1.00 |  | 48 (59.3) | 1.00 |  | 48 (52.7) | 1.00 |  |
| **AG** | 95 (30.8) | 85 (32.4) | 1.11 (0.77–1.62) | 0.57 | 23 (25.6) | 0.83 (0.48–1.45) | 0.52 | 25 (30.9) | 1.03 (0.59–1.78) | 0.93 | 37 (40.7) | 1.51 (0.91–2.52) | 0.11 |
| **GG** | 31 (10.1) | 25 (9.5) | 0.94 (0.53–1.67) | 0.83 | 11 (12.2) | 1.12 (0.52–2.38) | 0.78 | 8 (9.9) | 0.91 (0.39–2.12) | 0.82 | 6 (6.6) | 0.73 (0.29–1.87) | 0.51 |
| ***CYP3A5* c.219-237G>A** |  |  |  |  |  |  |  |  |  |  |  |  |  |
| **GG** | 149 (50.7) | 125 (48.8) | 1.00 |  | 49 (52.7) | 1.00 |  | 41 (48.8) | 1.00 |  | 35 (44.3) | 1.00 |  |
| **GA** | 113 (38.4) | 106 (41.4) | 1.12 (0.78–1.62) | 0.54 | 33 (35.5) | 0.91 (0.54–1.51) | 0.71 | 33 (39.3) | 1.07 (0.63–1.81) | 0.81 | 40 (50.6) | 1.47 (0.87–2.49) | 0.15 |
| **AA** | 32 (10.9) | 25 (9.8) | 0.98 (0.54–1.78) | 0.95 | 11 (11.8) | 1.10 (0.51–2.38) | 0.81 | 10 (11.9) | 1.21 (0.54–2.71) | 0.64 | 4 (5.1) | 0.55 (0.18–1.68) | 0.30 |
| ***GSTM1*** |  |  |  |  |  |  |  |  |  |  |  |  |  |
| **Non-null** | 203 (59.4) | 164 (56.6) | 1.00 |  | 63 (62.4) | 1.00 |  | 46 (52.9) | 1.00 |  | 55 (53.9) | 1.00 |  |
| **Null** | 139 (40.6) | 126 (43.4) | 1.19 (0.86–1.65) | 0.30 | 38 (37.6) | 0.90 (0.57–1.44) | 0.66 | 41 (47.1) | 1.39 (0.86–2.24) | 0.18 | 47 (46.1) | 1.35 (0.86–2.11) | 0.20 |
| ***GSTT1*** |  |  |  |  |  |  |  |  |  |  |  |  |  |
| **Non-null** | 257 (75.1) | 215 (74.1) | 1.00 |  | 73 (72.3) | 1.00 |  | 65 (74.7) | 1.00 |  | 77 (75.5) | 1.00 |  |
| **Null** | 85 (24.9) | 75 (25.9) | 1.14 (0.78–1.65) | 0.50 | 28 (27.7) | 1.30 (0.78–2.16) | 0.32 | 22 (25.3) | 1.11 (0.64–1.92) | 0.72 | 25 (24.5) | 1.03 (0.60–1.75) | 0.92 |
| ***SULT1A1* c.638G>A** |  |  |  |  |  |  |  |  |  |  |  |  |  |
| **GG** | 192 (47.5) | 155 (48.0) | 1.00 |  | 58 (52.7) | 1.00 |  | 51 (49.5) | 1.00 |  | 46 (41.8) | 1.00 |  |
| **GA** | 170 (42.1) | 143 (44.3) | 1.06 (0.77–1.45) | 0.73 | 49 (44.5) | 0.97 (0.62–1.50) | 0.88 | 43 (41.7) | 0.96 (0.61–1.52) | 0.86 | 51 (46.4) | 1.29 (0.82–2.04) | 0.27 |
| **AA** | 42 (10.4) | 25 (7.7) | 0.71 (0.41–1.23) | 0.23 | 3 (2.7) | **0.24 (0.07–0.81)** | **0.02** | 9 (8.7) | 0.78 (0.35–1.73) | 0.54 | 13 (11.8) | 1.23 (0.60–2.51) | 0.57 |
| ***SULT1A1* c.667A>G** |  |  |  |  |  |  |  |  |  |  |  |  |  |
| **AA** | 238 (58.9) | 232 (71.8) | 1.00 |  | 70 (63.6) | 1.00 |  | 77 (74.8) | 1.00 |  | 85 (77.3) | 1.00 |  |
| **AG** | 161 (39.9) | 86 (26.6) | 0.52 (0.38–0.73) | < 0.001* | 39 (35.5) | 0.75 (0.48–1.17) | 0.21 | 23 (22.3) | 0.41 (0.25–0.69) | 0.001* | 24 (21.8) | 0.40 (0.24–0.66) | < 0.001* |
| **GG** | 5 (1.2) | 5 (1.5) | 1.03 (0.29–3.70) | 0.96 | 1 (0.9) | 0.63 (0.07–5.65) | 0.68 | 3 (2.9) | 1.78 (0.41–7.74) | 0.44 | 1 (0.9) | 0.46 (0.05–4.06) | 0.49 |

ALL, acute lymphoblastic leukemia; AML, acute myeloid leukemia; aOR, adjusted odds ratio; CI, confidence intervals.

^a^ The age strata in AML is not relevant as it is for ALL, therefore, the AML cases have not been sub-divided by age.

^b^ aOR, odds ratio adjusted by skin color.

* Statistically significant (p Value < 0.01) after Bonferroni correction.
