# Supplementary material for: Polymorphisms in CYP1B1, CYP3A5, GSTT1, and SULT1A1 Are Associated with Early Age Acute Leukemia
Source: PLoS One. 2015 May 18;10(5):e0127308. doi: 10.1371/journal.pone.0127308 (PMC4436276; doi:10.1371/journal.pone.0127308)
Supplement: S4 Table — (DOC) [file pone.0127308.s004.doc]

**S4 Table. Genotype frequencies of *CYP3A4* and *SULT1A1* in males and early age acute leukemia, Brazil, 2000-2012.**

| **Genotypes** | **Controls** | **iALL ^b^** | **aOR (95% CI) ^a^** | ***p* Value** | **ALL ^c^** | **aOR (95% CI) ^a^** | ***p* Value** | **AML** | **aOR (95% CI) ^a^** | ***p* Value** |
| --- | --- | --- | --- | --- | --- | --- | --- | --- | --- | --- |
| ***CYP3A4* c.-392A>G** |  |  |  |  |  |  |  |  |  |  |
| **AA** | 104 (60.1) | 35 (71.4) | 1.00 |  | 27 (56.2) | 1.00 |  | 22 (44.9) | 1.00 |  |
| **AG** | 47 (27.2) | 8 (16.3) | 0.57 (0.24–1.35) | 0.20 | 18 (37.5) | 1.63 (0.81–3.29) | 0.17 | 25 (51.0) | 2.81 (1.40–5.64) | 0.004* |
| **GG** | 22 (12.7) | 6 (12.2) | 0.78 (0.29–2.10) | 0.62 | 3 (6.2) | 0.49 (0.14–1.77) | 0.28 | 2 (4.1) | 0.41 (0.09–1.92) | 0.26 |
| ***SULT1A1* c.667A>G** |  |  |  |  |  |  |  |  |  |  |
| **AA** | 138 (62.2) | 39 (63.9) | 1.00 |  | 39 (69.6) | 1.00 |  | 51 (83.6) | 1.00 |  |
| **AG** | 83 (37.4) | 21 (34.4) | 0.78 (0.42–1.43) | 0.41 | 15 (26.8) | 0.60 (0.31–1.17) | 0.13 | 10 (16.4) | 0.28 (0.13–0.60) | 0.001* |
| **GG** | 1 (0.5) | 1 (1.6) | 3.76 (0.19–72.8) | 0.38 | 2 (3.6) | 6.78 (0.59–78.0) | 0.13 | 0 (0.0) |  |  |

ALL, acute lymphoblastic leukemia; AML, acute myeloid leukemia; aOR, adjusted odds ratio; CI, confidence intervals; iALL, infant ALL.

^a^Odds ratio adjusted by skin color.

^b^ infant ALL patients comprise children ≤ 12 months-old at diagnosis.

^c^ ALL patients 13-24 months-old at diagnosis.

* Statistically significant (p Value < 0.01) after Bonferroni correction.
