# Supplementary material for: Polymorphisms in CYP1B1, CYP3A5, GSTT1, and SULT1A1 Are Associated with Early Age Acute Leukemia
Source: PLoS One. 2015 May 18;10(5):e0127308. doi: 10.1371/journal.pone.0127308 (PMC4436276; doi:10.1371/journal.pone.0127308)
Supplement: S5 Table — (DOC) [file pone.0127308.s005.doc]

**S5 Table. Genotype frequencies of *CYP3A4* and *SULT1A1* in females and early age acute leukemia, Brazil, 2000-2012.**

| **Genotypes** | **Controls** | **iALL ^b^** | **aOR (95% CI) ^a^** | ***p* Value** | **ALL ^c^** | **aOR (95% CI) ^a^** | ***p* Value** | **AML** | **aOR (95% CI) ^a^** | ***p* Value** |
| --- | --- | --- | --- | --- | --- | --- | --- | --- | --- | --- |
| ***CYP3A4* c.-392A>G** |  |  |  |  |  |  |  |  |  |  |
| **AA** | 75 (56.8) | 21 (51.2) | 1.00 |  | 21 (63.6) | 1.00 |  | 26 (61.9) | 1.00 |  |
| **AG** | 48 (36.4) | 15 (36.6) | 1.16 (0.54–2.49) | 0.71 | 7 (21.2) | 0.51 (0.20–1.32) | 0.17 | 12 (28.6) | 0.68 (0.31–1.51) | 0.35 |
| **GG** | 9 (6.8) | 5 (12.2) | 1.84 (0.54–6.26) | 0.33 | 5 (15.2) | 1.93 (0.56–6.63) | 0.29 | 4 (9.5) | 1.11 (0.30–4.14) | 0.88 |
| ***SULT1A1* c.667A>G** |  |  |  |  |  |  |  |  |  |  |
| **AA** | 97 (55.1) | 31 (63.3) | 1.00 |  | 38 (80.9) | 1.00 |  | 34 (69.4) | 1.00 |  |
| **AG** | 75 (42.6) | 18 (36.7) | 0.71 (0.37–1.38) | 0.32 | 8 (17.0) | 0.26 (0.11–0.59) | <0.01* | 14 (28.6) | 0.51 (0.26–1.03) | 0.06 |
| **GG** | 4 (2.3) | 0 (0.0) |  |  | 1 (2.1) | 0.64 (0.07–6.03) | 0.70 | 1 (2.0) | 0.65 (0.07–6.01) | 0.70 |

ALL, acute lymphoblastic leukemia; AML, acute myeloid leukemia; aOR, adjusted odds ratio; CI, confidence intervals; iALL, infant ALL.

^a^Odds ratio adjusted by skin color.

^b^ infant ALL patients comprise children ≤ 12 months-old at diagnosis.

^c^ ALL patients 13-24 months-old at diagnosis.

* Statistically significant (p Value < 0.01) after Bonferroni correction.
