# Supplementary material for: Polymorphisms in CYP1B1, CYP3A5, GSTT1, and SULT1A1 Are Associated with Early Age Acute Leukemia
Source: PLoS One. 2015 May 18;10(5):e0127308. doi: 10.1371/journal.pone.0127308 (PMC4436276; doi:10.1371/journal.pone.0127308)
Supplement: S6 Table — (DOC) [file pone.0127308.s006.doc]

**S6 Table. Combination of genotype frequencies of *CYP1B1*, *CYP3A4*, *CYP3A5*, *GSTT1, GSTM1* and *SULT1A1* and leukemia subtypes, Brazil, 2000-2012.**

| **Leukemia Subtype** | **Genetic Polymorphisms^a^** | ***CYP1B1*** **c.1294C>G** | | ***CYP3A4*** **c.-392A>G** | | ***CYP3A5*** **c.219-237G>A** | |
| --- | --- | --- | --- | --- | --- | --- | --- |
|  |  | **aOR (95% CI)^b^** | ***p* Value** | **aOR (95% CI)^b^** | ***p* Value** | **aOR (95% CI)^b^** | ***p* Value** |
| **iALL ^c^** | *GSTM1* | 0.82 (0.35–1.96) | 0.66 | 0.87 (0.40–1.87) | 0.71 | 1.04 (0.52–2.11) | 0.91 |
|  | *GSTT1* | 1.27 (0.54–2.97) | 0.58 | 1.79 (0.76–4.22) | 0.19 | 1.34 (0.59–3.03) | 0.49 |
|  | *SULT1A1* c.638G>A | 2.15 (0.70–6.58) | 0.18 | 0.77 (0.37–1.63) | 0.50 | 0.87 (0.43–1.75) | 0.69 |
|  | *SULT1A1* c.667A>G | 0.97 (0.43–2.18) | 0.94 | 0.76 (0.35–1.65) | 0.49 | 1.07 (0.55–2.06) | 0.85 |
| **ALL ^d^** | *GSTM1* | 1.62 (0.64–4.11) | 0.31 | 1.08 (0.46–2.54) | 0.86 | 1.50 (0.73–3.08) | 0.27 |
|  | *GSTT1* | 1.18 (0.46–3.01) | 0.74 | 1.31 (0.50–3.42) | 0.58 | 1.50 (0.66–3.45) | 0.34 |
|  | *SULT1A1* c.638G>A | 0.87 (0.40–1.93) | 0.74 | 1.02 (0.48–2.16) | 0.96 | 1.12 (0.56–2.24) | 0.75 |
|  | *SULT1A1* c.667A>G | 0.60 (0.29–1.25) | 0.17 | 0.33 (0.12–0.90) | 0.03 | 0.42 (0.18–0.96) | 0.04 |
| **AML** | *GSTM1* | 0.89 (0.40–1.97) | 0.77 | 1.73 (0.86–3.51) | 0.13 | 1.84 (0.85–3.99) | 0.12 |
|  | *GSTT1* | 0.67 (0.31–1.46) | 0.31 | 2.10 (0.88–4.97) | 0.09 | 1.46 (0.63–3.40) | 0.38 |
|  | *SULT1A1* c.638G>A | 0.91 (0.42–1.98) | 0.81 | 2.06 (0.96–4.42) | 0.06 | 1.93 (0.84–4.46) | 0.12 |
|  | *SULT1A1* c.667A>G | 0.24 (0.10–0.55) | 0.001* | 0.75 (0.36–1.55) | 0.43 | 0.66 (0.31–1.42) | 0.29 |

iALL, infant acute lymphoblastic leukaemia, ALL, acute lymphoblastic leukemia; AML, acute myeloid leukemia; aOR, adjusted Odds Ratio; CI, confidence intervals.

^a^ Comparison of homozygous wild-type versus heterozygous and homozygous variants for both polymorphisms.

^b^ Odds Ratio adjusted by ethnicity.

^c^ infant ALL patients comprise children ≤ 12 months-old at diagnosis.

^d^ ALL patients 13-24 months-old at diagnosis.

* Statistically significant (*p* Value < 0.01) after Bonferroni correction.
