# Supplementary material for: Polymorphisms in CYP1B1, CYP3A5, GSTT1, and SULT1A1 Are Associated with Early Age Acute Leukemia
Source: PLoS One. 2015 May 18;10(5):e0127308. doi: 10.1371/journal.pone.0127308 (PMC4436276; doi:10.1371/journal.pone.0127308)
Supplement: S7 Table — (DOC) [file pone.0127308.s007.doc]

**S7 Table. The genotype risk association in acute leukemia with different *MLL* status, Brazil, 2000-2012.**

|  |  | **Ctrl** | **Overall Cases *MLL*-GL** | | | **Overall Cases *MLL*-r** | | | **ALL *MLL*-GL** | | | **ALL *MLL*-r** | | | **AML *MLL*-GL** | | | **AML *MLL*-r** | | |
| --- | --- | --- | --- | --- | --- | --- | --- | --- | --- | --- | --- | --- | --- | --- | --- | --- | --- | --- | --- | --- |
|  |  | ***n*** | ***n*** | **aOR (95% CI)^a^** | ***p* Value** | ***n*** | **aOR (95% CI)^a^** | ***p* Value** | ***n*** | **aOR (95% CI)^a^** | ***p* Value** | ***n*** | **aOR (95% CI)^a^** | ***p* Value** | ***n*** | **aOR (95% CI)^a^** | ***p* Value** | ***n*** | **aOR (95% CI)^a^** | ***p* Value** |
| ***CYP1B1* c.1294C>G** | |  |  |  |  |  |  |  |  |  |  |  |  |  |  |  |  |  |  |  |
|  | **CC** | _78 | 31 | 1.00 |  | 32 | 1.00 |  | 14 | 1.00 |  | 22 | 1.00 |  | 17 | 1.00 |  | 10 | 1.00 |  |
|  | **CG** | 161 | 73 | 1.11 (0.67–1.84) | 0.70 | 46 | 0.67 (0.39–1.14) | 0.14 | 46 | 1.55 (0.80–3.02) | 0.19 | 34 | 0.73 (0.40–1.34) | 0.31 | 27 | 0.74 (0.38–1.45) | 0.38 | 12 | 0.54 (0.22–1.32) | 0.18 |
|  | **GG** | _78 | 22 | 0.73 (0.38–1.39) | 0.33 | 26 | 0.78 (0.42–1.46) | 0.45 | 17 | 1.22 (0.55–2.70) | 0.63 | 21 | 1.01 (0.50–2.02) | 0.98 | 5 | 0.31 (0.11–0.90) | 0.03 | 5 | 0.33 (0.10–1.13) | 0.08 |
| ***CYP3A4* c.-392A>G** | |  |  |  |  |  |  |  |  |  |  |  |  |  |  |  |  |  |  |  |
|  | **AA** | 182 | 68 | 1.00 |  | 68 | 1.00 |  | 45 | 1.00 |  | 50 | 1.00 |  | 23 | 1.00 |  | 18 | 1.00 |  |
|  | **AG** | _95 | 45 | 1.34 (0.85–2.13) | 0.21 | 30 | 0.86 (0.52–1.43) | 0.56 | 20 | 0.88 (0.49–1.60) | 0.68 | 22 | 0.89 (0.51–1.58) | 0.70 | 25 | 2.26 (1.20–4.24) | 0.01* | 8 | 0.74 (0.30–1.85) | 0.52 |
|  | **GG** | _31 | 14 | 1.18 (0.59–2.38) | 0.64 | 10 | 0.83 (0.39–1.80) | 0.64 | 11 | 1.36 (0.63–2.93) | 0.44 | 8 | 0.91 (0.39–2.11) | 0.82 | 3 | 0.78 (0.22–2.80) | 0.71 | 2 | 0.63 (0.14–2.85) | 0.54 |
| ***CYP3A5* c.219-237G>A** | |  |  |  |  |  |  |  |  |  |  |  |  |  |  |  |  |  |  |  |
|  | **GG** | 149 | 56 | 1.00 |  | 54 | 1.00 |  | 40 | 1.00 |  | 41 | 1.00 |  | 16 | 1.00 |  | 13 | 1.00 |  |
|  | **GA** | 113 | 55 | 1.32 (0.83–2.09) | 0.23 | 43 | 1.04 (0.64–1.67) | 0.88 | 29 | 0.97 (0.56–1.67) | 0.91 | 31 | 1.02 (0.60–1.73) | 0.96 | 26 | **2.21 (1.12–4.35)** | **0.02** | 12 | 1.07 (0.46–2.49) | 0.89 |
|  | **AA** | _32 | 13 | 1.18 (0.57–2.45) | 0.66 | 9 | 0.81 (0.35–1.84) | 0.62 | 12 | 1.52 (0.71–3.27) | 0.29 | 8 | 0.95 (0.40–2.24) | 0.90 | 1 | 0.32 (0.04–2.49) | 0.27 | 1 | 0.36 (0.05–2.91) | 0.34 |
| ***GSTM1*** | |  |  |  |  |  |  |  |  |  |  |  |  |  |  |  |  |  |  |  |
|  | **Non-null** | 203 | 74 | 1.00 |  | 70 | 1.00 |  | 47 | 1.00 |  | 51 | 1.00 |  | 27 | 1.00 |  | 19 | 1.00 |  |
|  | **Null** | 139 | 65 | 1.35 (0.90–2.02) | 0.15 | 48 | 1.07 (0.69–1.65) | 0.76 | 38 | 1.24 (0.76–2.02) | 0.38 | 35 | 1.04 (0.64–1.69) | 0.89 | 27 | 1.52 (0.85–2.71) | 0.16 | 13 | 1.15 (0.54–2.44) | 0.71 |
| ***GSTT1*** | |  |  |  |  |  |  |  |  |  |  |  |  |  |  |  |  |  |  |  |
|  | **Non-null** | 257 | 98 | 1.00 |  | 91 | 1.00 |  | 57 | 1.00 |  | 67 | 1.00 |  | 41 | 1.00 |  | 24 | 1.00 |  |
|  | **Null** | _85 | 41 | 1.40 (0.89–2.20) | 0.14 | 27 | 0.95 (0.57–1.57) | 0.83 | 28 | 1.63 (0.97–2.75) | 0.07 | 19 | 0.95 (0.54–1.70) | 0.87 | 13 | 1.10 (0.56–2.18) | 0.78 | 8 | 0.91 (0.38–2.21) | 0.84 |
| ***SULT1A1* c.638G>A** | |  |  |  |  |  |  |  |  |  |  |  |  |  |  |  |  |  |  |  |
|  | **GG** | 192 | 71 | 1.00 |  | 61 | 1.00 |  | 49 | 1.00 |  | 46 | 1.00 |  | 22 | 1.00 |  | 15 | 1.00 |  |
|  | **GA** | 170 | 66 | 1.06 (0.71–1.59) | 0.77 | 59 | 1.12 (0.74–1.70) | 0.60 | 38 | 0.89 (0.55–1.43) | 0.62 | 43 | 1.07 (0.67–1.72) | 0.77 | 28 | 1.46 (0.80–2.66) | 0.22 | 16 | 1.30 (0.61–2.76) | 0.49 |
|  | **AA** | _42 | 14 | 0.91 (0.46–1.78) | 0.77 | 7 | 0.51 (0.22–1.21) | 0.13 | 5 | 0.46 (0.17–1.24) | 0.12 | 5 | 0.51 (0.19–1.39) | 0.19 | 9 | 2.01 (0.84–4.79) | 0.12 | 2 | 0.50 (0.11–2.33) | 0.38 |
| ***SULT1A1* c.667A>G** | |  |  |  |  |  |  |  |  |  |  |  |  |  |  |  |  |  |  |  |
|  | **AA** | 238 | 110 | 1.00 |  | 89 | 1.00 |  | 63 | 1.00 |  | 64 | 1.00 |  | 47 | 1.00 |  | 25 | 1.00 |  |
|  | **AG** | 161 | 37 | 0.46 (0.30–0.71) | < 0.001* | 37 | 0.58 (0.37–0.90) | 0.02 | 26 | 0.55 (0.33–0.92) | 0.02 | 29 | 0.61 (0.37–0.99) | 0.05 | 11 | 0.31 (0.16–0.63) | 0.001* | 8 | 0.50 (0.22–1.14) | 0.10 |
|  | **GG** | __5 | 4 | 1.74 (0.44–6.80) | 0.43 | 1 | 0.48 (0.06–4.24) | 0.51 | 3 | 2.32 (0.52–10.2) | 0.27 | 1 | 0.67 (0.08–5.95) | 0.72 | 1 | 0.80 (0.09–7.16) | 0.84 | 0 |  |  |

ALL, acute lymphoblastic leukemia; AML, acute myeloid leukemia; aOR, adjusted odds ratio; CI, confidence intervals; Ctrl, controls; *MLL*-GL, *MLL* germ line; *MLL*-r, *MLL* rearranged.

^a^ Odds ratio adjusted by skin color.

* Statistically significant (*p* Value < 0.01) after Bonferroni correction.
