# Supplementary material for: Polymorphisms in CYP1B1, CYP3A5, GSTT1, and SULT1A1 Are Associated with Early Age Acute Leukemia
Source: PLoS One. 2015 May 18;10(5):e0127308. doi: 10.1371/journal.pone.0127308 (PMC4436276; doi:10.1371/journal.pone.0127308)
Supplement: S8 Table — (DOC) [file pone.0127308.s008.doc]

**S8 Table. Genotype frequencies of *CYP1B1*, *CYP3A4*, *CYP3A5*, *GSTT1,* *GSTM1* and *SULT1A1* in mothers of cases and controls, Brazil, 2000-2012.**

| **Genotype** | | **No. of Mothers of Controls (*n* = 65)** | **No. of Mothers of Cases (*n* = 69)** | **aOR (95% CI) ^a^** | ***p* Value** |
| --- | --- | --- | --- | --- | --- |
| ***CYP1B1* c.1294C>G** | |  |  |  |  |
|  | **CC** | 14 (29.8) | 13 (24.5) | 1.00 |  |
|  | **CG** | 21 (44.7) | 29 (54.7) | 1.49 (0.58–3.81) | 0.41 |
|  | **GG** | 12 (25.5) | 11 (20.8) | 0.99 (0.32–3.01) | 0.98 |
| ***CYP3A4* c.-392A>G** | |  |  |  |  |
|  | **AA** | 36 (62.1) | 35 (60.3) | 1.00 |  |
|  | **AG** | 17 (29.3) | 17 (29.3) | 1.03 (0.45–2.33) | 0.95 |
|  | **GG** | 5 (8.6) | 6 (10.3) | 1.23 (0.35–4.42) | 0.75 |
| ***CYP3A5* c.219-237G>A** | |  |  |  |  |
|  | **GG** | 30 (50.8) | 26 (50.0) | 1.00 |  |
|  | **GA** | 20 (33.9) | 17 (32.7) | 0.98 (0.43–2.26) | 0.96 |
|  | **AA** | 9 (15.3) | 9 (17.3) | 1.15 (0.40–3.43) | 0.79 |
| ***GSTM1*** | |  |  |  |  |
|  | **Non-null** | 38 (70.4) | 33 (55.0) | 1.00 |  |
|  | **Null** | 16 (29.6) | 27 (45.0) | 1.94 (0.90–4.22) | 0.09 |
| ***GSTT1*** | |  |  |  |  |
|  | **Non-null** | 40 (74.1) | 53 (88.3) | 1.00 |  |
|  | **Null** | 14 (25.9) | 7 (11.7) | 0.38 (0.14–1.02) | 0.05 |
| ***SULT1A1* c.638G>A** | |  |  |  |  |
|  | **GG** | 25 (41.7) | 32 (49.2) | 1.00 |  |
|  | **GA** | 25 (41.7) | 31 (47.7) | 0.97 (0.46–2.04) | 0.93 |
|  | **AA** | 10 (16.7) | 2 (3.1) | **0.16 (0.03–0.78)** | **0.02** |
| ***SULT1A1* c.667A>G** | |  |  |  |  |
|  | **AA** | 41 (68.3) | 44 (67.7) | 1.00 |  |
|  | **AG** | 16 (26.7) | 20 (30.8) | 1.16 (0.53–2.55) | 0.70 |
|  | **GG** | 3 (5.0) | 1 (1.5) | 0.31 (0.03–3.11) | 0.36 |

aOR, adjusted odds ratio; CI, confidence intervals.

^a^ Odds ratio adjusted by skin color.
