# Supplementary material for: Polymorphisms in CYP1B1, CYP3A5, GSTT1, and SULT1A1 Are Associated with Early Age Acute Leukemia
Source: PLoS One. 2015 May 18;10(5):e0127308. doi: 10.1371/journal.pone.0127308 (PMC4436276; doi:10.1371/journal.pone.0127308)
Supplement: S9 Table — (DOC) [file pone.0127308.s009.doc]

**S9 Table. Mother-child paired genotypes, Brazil, 2000-2012.**

| **Child Genotype** | **Maternal Genotype** | | | | | |
| --- | --- | --- | --- | --- | --- | --- |
|  | **Homozygote Wild-type** | | | **Heterozygote/Homozygote Variant** | | |
|  | **Control/Case** | **aOR (95% CI) ^a^** | ***p* Value** | **Control/Case** | **aOR (95% CI) ^a^** | ***p* Value** |
| ***CYP1B1* c.1294C>G** |  |  |  |  |  |  |
| **CC** | 3/4 | 1.00 |  | 5/2 | 1.00 |  |
| **CG + GG** | 8/6 | 0.56 (0.90–3.52) | 0.66 | 16/24 | 3.75 (0.65–21.7) | 0.22 |
| ***CYP3A4* c.-392A>G** |  |  |  |  |  |  |
| **AA** | 16/17 | 1.00 |  | 6/2 | 1.00 |  |
| **AG + GG** | 7/6 | 0.81 (0.22–2.92) | 0.74 | 7/14 | 6.00 (0.95–37.8) | 0.09 |
| ***CYP3A5* c.219-237G>A** |  |  |  |  |  |  |
| **GG** | 10/16 | 1.00 |  | 5/6 | 1.00 |  |
| **GA + AA** | 4/2 | 0.31 (0.05–2.03) | 0.37 | 9/16 | 1.48 (0.35–6.26) | 0.72 |
| ***GSTM1*** |  |  |  |  |  |  |
| **Non-null** | 24/17 | 1.00 |  | 7/8 | 1.00 |  |
| **Null** | 11/10 | 1.28 (0.45–3.70) | 0.64 | 7/16 | 2.00 (0.52–7.70) | 0.31 |
| ***GSTT1*** |  |  |  |  |  |  |
| **Non-null** | 30/36 | 1.00 |  | 9/1 | 1.00 |  |
| **Null** | 7/9 | 1.07 (0.36–3.22) | 0.90 | 3/5 | **15.0 (1.22–185.0)** | **0.04** |
| ***SULT1A1* c.638G>A** |  |  |  |  |  |  |
| **GG** | 19/21 | 1.00 |  | 11/12 | 1.00 |  |
| **GA + AA** | 5/9 | 1.63 (0.46–5.73) | 0.45 | 24/18 | 0.69 (0.25–1.91) | 0.47 |
| ***SULT1A1* c.667A>G** |  |  |  |  |  |  |
| **AA** | 29/30 | 1.00 |  | 13/11 | 1.00 |  |
| **AG + GG** | 11/10 | 0.88 (0.32–2.38) | 0.80 | 6/9 | 1.77 (0.48–6.56) | 0.39 |

aOR, adjusted odds ratio; CI, confidence intervals.

^a^ Odds ratio adjusted by skin color.
