# Supplementary material for: Polymorphisms in CYP1B1, CYP3A5, GSTT1, and SULT1A1 Are Associated with Early Age Acute Leukemia
Source: PLoS One. 2015 May 18;10(5):e0127308. doi: 10.1371/journal.pone.0127308 (PMC4436276; doi:10.1371/journal.pone.0127308)
Supplement: S10 Table — (DOC) [file pone.0127308.s010.doc]

**S10 Table. Gene expression according to child genotype, Brazil, 2000-2012.**

| **Child Genotype ^a^** | **Overall cases** | | | **ALL** | | | **AML** | | |
| --- | --- | --- | --- | --- | --- | --- | --- | --- | --- |
|  | **Positive Expression ^b^**  ***n* (%)** | **Negative Expression ^b^**  ***n* (%)** | ***p* Value** | **Positive Expression ^b^**  ***n* (%)** | **Negative Expression ^b^**  ***n* (%)** | ***p* Value** | **Positive Expression ^b^**  ***n* (%)** | **Negative Expression ^b^**  ***n* (%)** | ***p* Value** |
| ***CYP1B1* c.1294C>G** |  |  |  |  |  |  |  |  |  |
| **Total** | 24 (27.6) | 63 (72.4) |  | 9 (17.3) | 43 (82.7) |  | 15 (42.9) | 20 (57.1) |  |
| **CC** | 11 (44.0) | 14 (56.0) |  | 3 (27.3) | 8 (72.7) |  | 8 (57.1) | 6 (42.9) |  |
| **CG** | 11 (26.2) | 31 (73.8) | 0.78 | 4 (17.4) | 19 (82.6) | 0.66 | 7 (36.8) | 12 (63.2) | 0.30 |
| **GG** | 2 (10.0) | 18 (90.0) | 0.18 | 2 (11.1) | 16 (88.9) | 0.34 | 0 (0.0) | 2 (100.0) | 0.47 |
| **CG + GG** | 13 (21.0) | 49 (79.0) | **0.04** | 6 (14.6) | 35 (85.4) | 0.38 | 7 (33.3) | 14 (66.7) | 0.19 |
| ***CYP3A5* c.219-237G>A** |  |  |  |  |  |  |  |  |  |
| **Total** | 13 (14.4) | 77 (85.6) |  | 4 (7.7) | 48 (92.3) |  | 9 (23.7) | 29 (76.3) |  |
| **GG** | 8 (20.5) | 31 (79.5) |  | 3 (12.5) | 21 (87.5) |  | 5 (33.3) | 10 (66.7) |  |
| **GA** | 3 (7.3) | 38 (92.7) | 0.11 | 1 (4.5) | 21 (95.5) | 0.61 | 2 (10.5) | 17 (89.5) | 0.20 |
| **AA** | 2 (20.0) | 8 (80.0) | 1.00 | 0 (0.0) | 6 (100.0) | 1.00 | 2 (50.0) | 2 (50.0) | 0.60 |
| **GA + AA** | 5 (9.8) | 46 (90.2) | 0.23 | 1 (3.6) | 27 (96.4) | 0.32 | 4 (10.6) | 19 (50.0) | 0.44 |

ALL, acute lymphoblastic leukemia; AML, acute myeloid leukemia.

^a^ *CYP3A4* gene expression has been evaluated, however no expression has been detected in any of the tested samples (*n* = 40).

^b^ Expression status was defined as “positive” whenever mRNA of the specified gene was identified or “negative” in case mRNA levels were not detected.
